# Response to pazopanib‐based combination regimen in a case of FGFR3 amplified gastric adenocarcinoma

**DOI:** 10.1002/ccr3.4986

**Published:** 2021-11-07

**Authors:** Sewanti Limaye, Darshana Patil, Dadasaheb Akolkar, Navin Srivastava, Revati Patil, Sachin Apurwa, Sanket Patil, Jinumary John, Rahul Gosavi, Prabhu Nesargikar, Prashant Kumar, Vineet Datta, Chirantan Bose, Zarrine Raazi, Ajay Srinivasan, Rajan Datar

**Affiliations:** ^1^ Kokilaben Dhirubhai Ambani Hospital and Medical Research Institute Mumbai Maharashtra India; ^2^ Datar Cancer Genetics Nasik Maharashtra India; ^3^ HCG Cancer Centre Bengaluru Karnataka India; ^4^ Institute of Bioinformatics Bengaluru Karnataka India; ^5^ Manipal Academy of Higher Education Manipal Karnataka India; ^6^ Somaiya Institute of Research and Consultancy Somaiya Vidyavihar University Mumbai Maharashtra India

**Keywords:** combination regimen, encyclopedic tumor analysis, gastric adenocarcinoma, pazopanib, personalized cancer treatment, precision oncology, stomach cancer

## Abstract

Angiogenesis inhibitors (AGI) are not presently used for the treatment of gastric cancers. This report demonstrates that angiogenesis inhibitor can be safely and effectively used in combination with cytotoxic anti‐cancer agents for treatment of Gastric cancers.

## Graphical abstract

This case of Gastric Adenocarcinoma had progressed following failure of several prior lines of standard of care regimens. Multi‐analyte profiling of freshly biopsied tumor sample indicated FGFR3 amplification along with sensitivity of tumor cells towards various chemotherapy drugs. The patient received personalized combination regimen of Pazopanib with Gemcitabine and Pemetrexed which yielded significant regression of metastatic deposits.

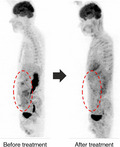

## 1 INTRODUCTION

Gastric cancer ranks 5th in terms of incidence and third in terms of mortality globally, with more than 1 million new cases and nearly 800,000 deaths annually.^1^ Most gastric cancers are diagnosed at advanced stages due to late symptomatic manifestations including nausea, vomiting, anorexia, weight loss, and ascites.^2^, ^3^ Standard of care systemic treatment options in gastric cancers especially adenocarcinomas (AD) include combinations of 5‐Fluorouracil, Platins, Taxanes, and Irinotecan which remain the mainstay of systemic treatments and are used in a perioperative setting despite the advent of various targeted agents and immune checkpoint inhibitors (ICI).^4^, ^5^, ^6^ Targeted agents which are used in treatment if gastric AD include Trastuzumab, based on HER2 status determined by IHC and Larotrectinib (as part of labeled pan‐solid tumor indication) in addition to ICIs such as Nivolumab (based on CPS), Pembrolizumab (based on MSI/MMR status). Angiogenesis inhibitors have remained largely absent in treatment of gastric cancers until the approval of Ramucirumab, a monoclonal antibody (mAb) which targets VEGFR2.^4^, ^7^, ^8^

Fibroblast growth factor receptors (FGFR) are being studied as potential targets in varied different kinds of solid tumors including gastric cancers.^9^ FGFR is a transmembrane receptor family with four members (FGFR 1–4) that bind to fibroblast growth factors (FGFs),^10^ and play important developmental roles, from early embryogenesis to organ formation. A study of 4,853 tumors by next‐generation sequencing (NGS) identified aberrant FGFR3 in 22% of urothelial carcinomas (UC), 4% of glioma, 3% of carcinoma of unknown primary and endometrial carcinoma, 2% of pancreatic, ovarian, and gastric carcinomas.^11^ A recent study identified the FGFR3/AKT axis as an escape pathway responsible for trastuzumab resistance in gastric cancer, thus indicating the inhibition of FGFR3 as a potential strategy to modulate this resistance.^12^ Pazopanib is a non‐selective multi‐targeted kinase inhibitor with inhibitory effects on FGFR as well as VGEF (vascular endothelial growth factor), PEGF (platelet endothelial growth factor), and stem‐cell factor c‐kit. Pazopnib has been previously evaluated for treatment of hormone positive, endocrine‐resistant metastatic breast cancer with FGFR1 amplifications,^13^ as well as in metastatic urothelial carcinoma with FGFR amplifications.^14^ Pazopanib is the Standard of Care treatment (labeled indication) for Renal Cell Carcinomas, Soft Tissue Sarcomas, and Gastrointestinal Stromal Tumors (GIST).^15^ Pazopanib has been tested with combination chemotherapy in advanced gastric cancer with promising results.^16^

Here, we report a case of gastric cancer that responded favorably to FGFR3 targeting with Pazopanib in combination with chemotherapy agents based on multi‐analyte the tumor profiling.^17^

## 2 CASE PRESENTATION

A 55‐year‐old male patient presented with anorexia and icterus. Upper gastrointestinal scopy revealed gastric and duodenal ulcers with gastric obstruction. HPE of biopsied tumor tissue indicated gastric adenocarcinoma (AD). Diagnostic laparoscopy showed omental and peritoneal deposits which were confirmed by histopathological examination (HPE) as metastases. HER2 status was not initially evaluated by Immunohistochemistry. The patient received neoadjuvant Standard of Care (SoC) chemotherapy with Docetaxel, Cisplatin, and 5‐Fluorouracil along with 50 Gy/28 days of radiotherapy. Post‐chemoradiotherapy, PET‐CT showed good response to the treatment with reduced wall thickening in stomach. The patient subsequently underwent Radical Distal Gastrectomy, with Roux‐En‐Y reconstruction. Pathologically determined stage was pT3N0 of a moderately differentiated AD. The patient received adjuvant 50.4 Gy #28 CTRT radiation therapy along with oral Tab Capecitabine till April 2018. Follow‐up PET‐CT scan after six months indicated deposits along the transverse colon and in the pelvic peritoneum along with ascites.

**FIGURE 1:**
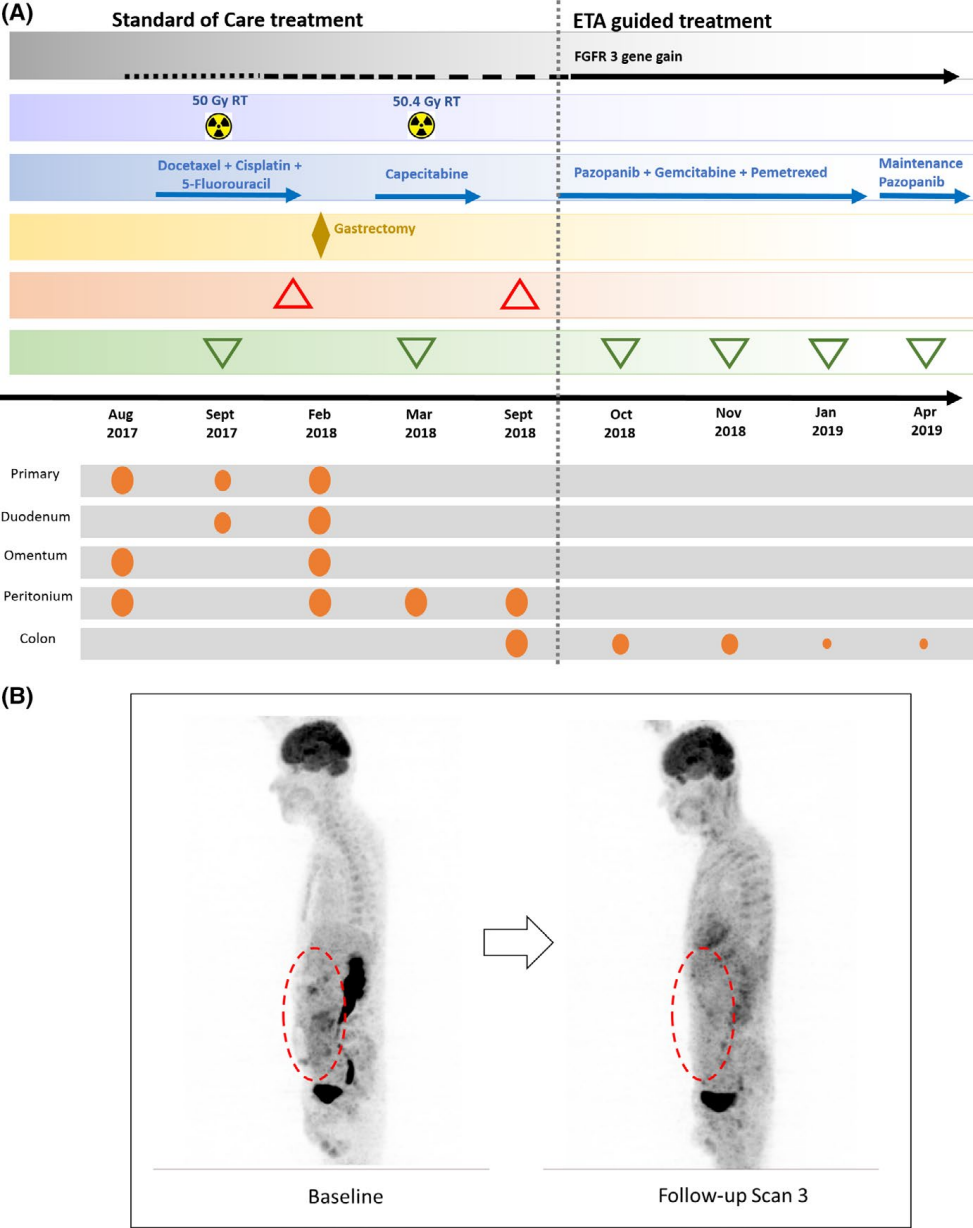
(A) Clinical timeline of 55‐year‐old male patient with FGFR3 gene amplification in gastric cancer; ♦ locoregional treatment; ▲ progression/recurrence; ▼ treatment response/regression; ⬮ presence of malignant mass at various sites; (B) PET‐CT scan‐ Baseline Vs Partial Response

Due to disease progression, patient underwent a biopsy to obtain fresh tumor tissue which was used for multi‐analyte Encyclopedic Tumor Analysis (ETA).^17^ NGS profiling of tumor tissue DNA identified mutations in NOTCH3 (p.G2218G), ATM (c.1236‐2A>T; intronic), and GNAS (p.R201C), in addition to amplification of FGFR3. In vitro chemoresponse profiling of viable tumor cells indicated sensitivity toward Gemcitabine, Pemetrexed, Doxorubicin, Topotecan, and Epirubicin (in descending order). HER2 amplifications were not detected in NGS and HER2 status by IHC was not ascertainable due to insufficient biopsy (patient was unable to undergo a repeat biopsy). Prior FFPE blocks/slides were unavailable for evaluation. Based on ETA findings, the patient was then treated with a combination of Tab Pazopanib (400 mg, PO, 1 OD), IV Gemcitabine (800 mg, D1 and D8 of 21 Day cycle), and IV Pemetrexed (400 mg, D1 and D8 of 21 Day cycle). The patient received 5 cycles of the combination regimen between October 2018 and February 2019. The patient underwent 4 follow‐up radiological imaging scans between October 2018 and April 2019. Significant response to treatment was observed at day 28 as well as all subsequent imaging scans, with no new lesions. Thereafter, the patient continued to receive maintenance treatment with Oral Tab Pazopanib. The patient tolerated the treatment regimen well with minimal and manageable profile of adverse events (AE), which included one incidence each of Grade III Fatigue, Thrombocytopenia, Sepsis, and Pneumothorax. All AEs resolved within a week and did not necessitate treatment suspension or dose modification. There were no Grade IV AEs. Patient‐reported significant improvement in general health and reduction of symptoms. The patient continued the maintenance regimen for several months but declined further follow‐up or imaging. Unfortunately, the patient passed away in March 2020 at the beginning of the COVID pandemic. A clinical timeline of the patient, including all the received treatments, is shown in Figure 1A, along with the PET‐CT scan in Figure 1B, showing a significant response to the ETA‐guided drug treatment.

## 3 DISCUSSION

The Cancer Genome Atlas (TCGA) dataset manifests 432 cases of gastric cancer (SC) harboring FGFR3 amplification wherein 24 cases showed copy number gain and 12 cases with copy number loss. A prior study reported a patient diagnosed with urothelial cancer with amplification of FGFR3 (11 copies), CCND1 (21 copies), and FGF19 (21 copies), and FGFR3 p.(S249C) mutation with estimated mutant allele frequency of 58%. The patient was treated with pazopanib and showed durable partial response.^14^ Kim et al. reported a case of metastatic gastric cancer harboring a novel FGFR3‐TACC3 fusion which has been rarely reported in gastrointestinal cancer.^18^ In the present case, profiling of tumor DNA identified FGFR3 amplification with 10 copies. Pazopanib is widely known to exert its action against VEGFR1, VEGFR2, VEGFR3, PDGFRα and β, FGFR1, FGFR3, c‐KIT, LCK, and macrophage colony‐stimulating factor‐1 receptor.^19^, ^20^, ^21^, ^22^ FGFR2 expression has also been indicated as a potential predictor of treatment response in advanced gastric cancer patients who have been treated with Pazopanib in combination with Capecitabine and Oxaliplatin.^16^ In a phase II trial, Dovitinib an FGFR inhibitor was efficacious against FGFR3‐mutated or overexpressed urothelial cancer.^23^ The FiGhTeR trial is a phase II, single‐arm, open‐label study to assess safety and activity of the FGFR inhibitor pemigatinib as second‐line treatment strategy in metastatic EGJ/GC patients progressing under trastuzumab‐containing therapies.^24^ Pazopanib is approved for use in advanced renal cell cancer and soft tissue sarcoma patients who have received prior chemotherapy. Under the Phase II study of the Arbeitsgemeinschaft internistische Onkologie (AIO) trial, pazopanib in combination with 5‐fluorouracil, leucovorin, and oxaliplatin showed promising outcome in advanced gastric cancer.^25^ In a Phase II trial, first‐line Pazopanib plus fluoropyrimidine and platinum in gastric cancer yielded OS of 10.1–10.5 months.^26^

Although Trastuzumab is SoC in gastric cancers with HER2 overexpression (IHC), the same could not be evaluated in this patient due to sample insufficiency. This conundrum is often experienced in the clinical setting where IHC analysis of relevant biomarkers is impeded due to sample insufficiency or non‐representative sampling. Though HER2 amplification was not observed on NGS, and HER2 status could not be ascertained on IHC, NGS profiling successfully identified an alternate therapeutically targetable indication, that is, FGFR. Patient showed sustained response to treatment over the treatment duration leading to significant regression of the metastatic lesions.

The combination of Gemcitabine and Pemetrexed has been evaluated in Advanced Non‐Small Cell lung Cancer with myelosuppression, especially neutropenia, as a significant adverse event.^27^, ^28^ In the present case, apart from transient Grade III AEs, there were no significant or myelosuppressive AEs indicating the safety of the treatment regimen.

## 4 CONCLUSION

In the current study, we report a case of Gastric AD with FGFR3 gene amplification (10 copies), where treatment with Pazopanib in combination with Gemcitabine and Pemetrexed yielded partial response. Further studies may be necessary to establish the viability of such combination regimens in FGFR3 amplified gastric cancer patients.

## CONFLICT OF INTEREST

SL, PN, and PK have no potential conflicts of interest to declare. DP, DA, NS, RP, SA, SP, JJ, RG, VD, CB, ZR, and AS are in full‐time employment of the Study Sponsor who offers commercial services in oncology. RD is the founder and CMD of the Study Sponsor.

## AUTHOR CONTRIBUTIONS

Sewanti Limaye: Study Design, Study Supervision, and Manuscript Writing. Darshana Patil and Ajay Srinivasan: Study Design, Study Supervision, Data Analysis, and Manuscript Review. Dadasaheb Akolkar and Vineet Datta: Study Design, Study Supervision, and Data Analysis. Navin Srivastava and Revati Patil: Study Design, Sample Analysis, and Data Analysis. Sachin Apurwa: Bioinformatics Analysis and Data Review. Sanket Patil: Sample Analysis and Data Analysis. Jinumary John and Rahul Gosavi: Data Review and Reporting. Prabhu Nesargikar: Data Analysis and Data Review. Prashant Kumar and Chirantan Bose: Data Review and Manuscript Writing. Zarrine Raazi: Bioinformatics Analysis and Manuscript Writing. Rajan Datar: Study Design, Study Supervision, Funding, Data Analysis, and Manuscript Review.

## CONSENT

The patient received personalized treatment as participant of the RESILIENT trial (CTRI/2018/02/011808), which was previously approved by the Institutional Ethics Committees of Datar Cancer Genetics as well as HCG Manavata Cancer Centre. The patient provided written Informed Consent prior to enrollment and sample collection. The patient also consented to publication of deidentified data and findings. The RESILIENT trial was conducted in accordance with existing Ethical and Regulatory considerations including the Declaration of Helsinki.

## Data Availability

Data may be made available to qualified researchers upon reasonable request.
